# Preparation and Mechanical Properties of UV-Curable Epoxy Acrylate/Modified Aramid Nanofiber Nanocomposite Films

**DOI:** 10.3390/nano13222960

**Published:** 2023-11-16

**Authors:** Ying Wang, Zhenxing Sun, Peixu Yin, Rongjun Qu, Ying Zhang, Changmei Sun

**Affiliations:** School of Chemistry and Materials Science, Ludong University, Yantai 264025, China; 15763519179@163.com (Z.S.); 18305486043@163.com (P.Y.); zhangying6613@163.com (Y.Z.); sunchangmei0535@126.com (C.S.)

**Keywords:** UV-curable, aramid nanofiber, epoxy acrylate, nanocomposite films, mechanical properties

## Abstract

In order to enhance the mechanical properties of UV-curable epoxy acrylate (EA)-based coatings, 3-(trimethoxysilyl)propyl methacrylate modified aramid nanofibers (T-ANFs) were synthesized and used as nanofillers to prepare EA/T-ANF nanocomposite films. The morphology of T-ANFs was characterized by transmission electron microscopy. The chemical structure of T-ANFs was analyzed via infrared spectroscopy, confirming successful grafting of methyl methacryloyloxy groups onto the surface of aramid nanofibers (ANFs). Real-time infrared spectroscopy was employed to investigate the influence of ANFs and T-ANFs on the photopolymerization kinetics of the EA film. The results revealed that the addition of ANFs and T-ANFs led to a decrease in the photopolymerization rate during the initial stage but had little impact on the final double bond conversion, with all samples exhibiting a conversion rate of over 83%. The incorporation of ANFs improved the tensile strength of the films while significantly reducing their Young’s modulus. In contrast, the addition of T-ANFs led to a substantial increase in both tensile stress and Young’s modulus of the films. For instance, the tensile strength and Young’s modulus of the 0.1 wt% of T-ANF film increased by 52.7% and 41.6%, respectively, compared to the pure EA film. To further study the dispersion morphology and reinforcement mechanism, the cross-sectional morphology of the films was characterized by scanning electron microscopy.

## 1. Introduction

Nanomaterials exhibit various special properties due to their nanoscale size, such as the unique surface effects, which make them important in the field of polymer reinforcement. As a one-dimensional nanomaterial, aramid nanofibers (ANFs) retain the high-temperature resistance, excellent mechanical properties, and low density of aramid fibers while possessing the high specific surface area of nanomaterials. Since they were first prepared by Kotov et al. [1] in 2011, ANFs have received widespread attention. ANFs have been extensively studied in the fields of battery separators, adsorption filtration, polymer reinforcement, thermal insulation, and flexible electronic devices. ANFs have been extensively studied for their potential to improve the mechanical properties of various polymers, including polyurethane [2,3,4], rubber [5,6,7,8], epoxy resin [9,10,11], polyvinyl alcohol [12], and polypyrrole [13]. For example, Wang et al. [6] found that adding 5 wt% of ANFs increased the tensile strength and tear strength of carboxylated acrylonitrile butadiene rubber by 182% and 101%, respectively. Guo et al. [13] combined the outstanding mechanical properties of ANFs with the conductivity of polypyrrole to prepare ANF/polypyrrole composite films with excellent electromagnetic shielding, wearable sensing, resistive heating, and photothermal conversion performance. ANFs are mainly connected to the polymer matrix through interface interactions such as hydrogen bonds. Introducing reactive functional groups on the surface of ANFs enables them to establish a tighter connection with the polymer matrix through covalent bonds, thereby further enhancing the mechanical properties of nanocomposites. For instance, Jung et al. [11] used 3-glycidoxypropyltrimethoxysilane as a modifier to prepare epoxy-functionalized ANFs, which increased the tensile strength and Young’s modulus of epoxy resin by 14% and 16.8%, respectively.

UV-curing technology is an efficient, energy-saving, and environmentally friendly coating preparation technique that has gained widespread attention since its inception. However, as a pure polymer-based coating, it often exhibits lower mechanical performance. The addition of nanofillers is an effective approach to enhance the performance of UV-curable coatings. For example, incorporating nanoscale oxides (such as silica [14,15], titanium dioxide [16], zinc oxide [17]), layered silicate clay (such as montmorillonite [18,19], kaolinite [20]), nanocarbon materials (such as graphene [21,22,23,24], carbon nanotubes [25]), and cage-like polyhedral oligomeric silsesquioxane [26,27,28,29] particles can effectively improve the coating properties. However, there are two common issues encountered in the preparation of UV-curable nanocomposites. Firstly, due to the large specific surface area of nanomaterials, they tend to agglomerate within the polymer matrix, which affects the coating performance. Secondly, nanomaterials can compete for absorption or act as barriers to UV light, leading to a decrease in the final double-bond conversion rate during the UV-curing reaction. In a previous study [30], we synthesized ANFs using a bottom-up approach and incorporated them into a UV-curable system to prepare nanocomposite films. The results showed an improvement in the film’s elongation at break and tensile strength, while the Young’s modulus decreased. This was mainly due to the aggregation of ANFs within the polymer matrix, leading to a deterioration in mechanical properties. Additionally, the connection between the ANFs and the polymer matrix was established only through hydrogen bonding and π-π conjugation, resulting in a decrease in the network crosslinking density and stiffness of the polymer.

**Figure 1:**
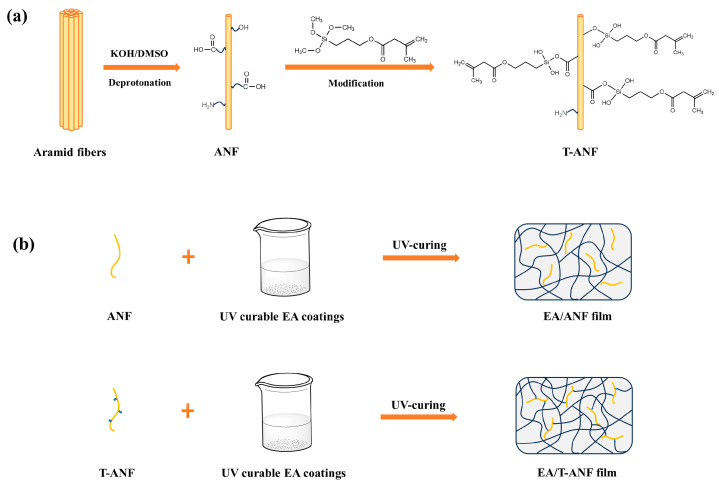
Synthetic mechanism of (**a**) T-ANF and (**b**) EA/ANF and EA/T-ANF nanocomposite films.

In this study, a top-down approach was employed to prepare ANFs. During the deprotonation process, carboxylic acid, amine, hydroxyl groups, and other functional groups were generated on the surface of ANF, enhancing its reactivity [11,31]. Furthermore, ANFs were first modified using 3-(trimethoxysilyl)propyl methacrylate (TMSPMA) to prepare TMSPMA-functionalized ANFs (T-ANFs), the synthetic mechanism is illustrated in Figure 1a. Different contents of ANFs and T-ANFs were separately added to UV-curable epoxy acrylate-based coatings to prepare nanocomposite films. The T-ANFs, due to the presence of methyl methacryloyloxy groups on their surface, were able to disperse more uniformly within the polymer matrix. Additionally, they could participate in the polymerization reaction under UV irradiation, forming a chemical bond with the polymer and enhancing the network crosslinking density and rigidity, as depicted in Figure 1b. The morphology and chemical structure of the T-ANFs were characterized by transmission electron microscopy (TEM) and flourier transform infrared spectroscopy (FTIR), respectively. The influence of ANFs and T-ANFs on the UV curing kinetics of the coatings was analyzed using real-time FTIR. The effects of ANFs and T-ANFs on the mechanical properties of the films were investigated through tensile testing, and the dispersion morphology of ANFs in the polymer and the interfacial interactions between ANFs and the polymer matrix were analyzed by scanning electron microscopy (SEM) imaging of the film cross-section.

## 2. Materials and Methods

### 2.1. Materials

Aramid fibers (Kevlar K49) were purchased from DuPont Company in the United States. Acetone was purchased from Yantai Yuandong Fine Chemical Co., Ltd. (Yantai, China). Anhydrous ethanol, KOH, and 3-(trimethoxysilyl)propyl methacrylate (TMSPMA) were all purchased from China Sinopharm Chemical Reagent Co., Ltd. (Shanghai, China) Dimethyl sulfoxide (DMSO) was purchased from Guangzhou Xilong Scientific Co., Ltd. (Guangzhou, China). Tri(propylene glycol) diacrylate (TPGDA) and epoxy acrylate (EA) were supplied by Allnex Resins Co., Ltd. (Shanghai, China) Benzophenone (BP) and triethanolamine (TEA) were respectively purchased from Tianjin Bodi Chemicals Co., Ltd., Tianjin, China, and Tianjin Regent Chemicals Co., Ltd., Tianjin, China.

### 2.2. Synthesis of ANFs

Before use, the aramid fibers were cut into pieces of less than 1 cm in length and put into a Soxhlet extractor. The fibers were washed with acetone and anhydrous ethanol for 12 h, respectively, then dried and kept for future use. An amount of 1.44 g of KOH and 400 mL of DMSO were added into a three-necked flask and stirred at 70 °C for 2 h until the KOH was completely dissolved. After cooling, 0.96 g of aramid fibers were added and mechanically stirred under nitrogen protection for 7 d, resulting in a dark red ANF/DMSO solution.

### 2.3. Synthesis of T-ANF

1 g of TMSPMA was added to a 250 mL ANF/DMSO solution and stirred at 80 °C for 24 h. After cooling, the modified ANFs were added to 250 mL of deionized water and evenly stirred. The mixture was centrifuged at 15,000 rpm for 20 min. The collected T-ANFs were washed with deionized water by centrifugation until the pH reached neutrality, followed by rinsing with acetone. The precipitate was collected and vacuum-dried at 60 °C to obtain T-ANFs.

### 2.4. Preparation of UV-Curable EA/T-ANF Nanocomposite Films

A certain amount of T-ANFs was added to 20 g of active diluent TPGDA and dispersed by ultrasonication for 15 min. Then, 16 g of oligomer EA, 1.08 g of photoinitiator BP, and 0.36 g of co-initiator TEA were added and uniformly stirred to obtain a UV-curable nanocomposite coating. The addition amounts of T-ANFs were 0.05 wt%, 0.1 wt%, 0.2 wt%, and 0.5 wt% of the total weight of the UV-curable coating, respectively, represented as EA/T-ANF-0.05, EA/T-ANF-0.1, EA/T-ANF-0.2, and EA/T-ANF-0.5. Then, the nanocomposite coating was uniformly applied onto glass substrates using a film applicator and cured by a ZB300 UV (Tai’an Zibor Photoelectric Technology Co., Ltd., Tai’an, China), curing equipment with a Fusion 300 s UV light source (the light intensity is 2.5 W/cm^2^) to obtain EA/T-ANF nanocomposite films. For comparison, a series of EA/ANF nanocomposite films were prepared with ANF addition amounts of 0.05 wt%, 0.1 wt%, and 0.2 wt% of the total weight of the UV-curable coating, denoted as EA/ANF-0.05, EA/ANF-0.1, and EA/ANF-0.2, respectively.

### 2.5. Characterization

TEM analysis was conducted using a 1400 Plus electron microscope (JEOL, Tokyo, Japan). ANFs and T-ANFs were dispersed in anhydrous ethanol and dropped onto copper grids for morphology observation.

FTIR was tested on a Nicolet iS50 spectrometer (Thermo Scientific, Waltham, MA, USA). The photopolymerization kinetics were analyzed using real-time FTIR. The coating was applied to a KBr crystal, and after a certain UV irradiation time, the infrared spectra were immediately measured using a UV point light source. The peak area data of the C=C double bond at 1636 cm^−1^ were obtained using EZ OMNIC8.2 software, and the conversion rate of the C=C double bond at different irradiation times was calculated using the following formula:

(1) $$C=100\times (1-\frac{A_{t}}{A_{0}})$$

where *A*_0_ and *A_t_* represent the peak area of the C=C double bond at *t* = 0 and *t* time, respectively.

Tensile tests were carried out using an Instron 5967 universal testing machine according to the -standard [32]. The cured film (with a thickness of approximately 0.075 mm) was cut into samples measuring 300 mm in length and 10mm in width. During the testing process, the gauge length was set at 100 mm, and the crosshead speed was 1 mm/min. Each sample was tested in parallel for 5 times.

The cross-sectional morphology of the films was observed using a TESCAN MIRA LMS SEM after liquid nitrogen fracturing and gold sputtering before testing.

## 3. Results and Discussion 

### 3.1. Characterization of T-ANFs

**Figure 2:**
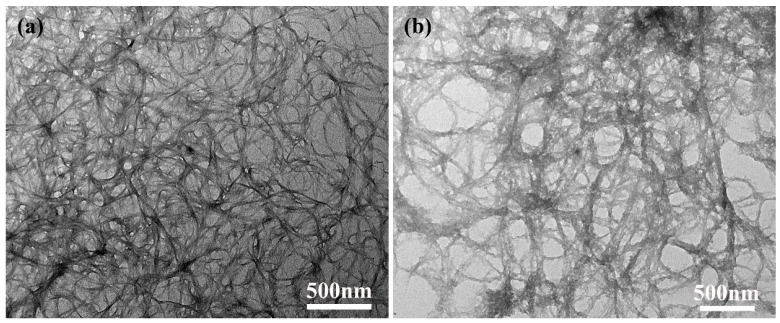
TEM images of the (**a**) ANFs and (**b**) T-ANFs.

The morphology of ANFs and T-ANFs was studied using TEM and shown in Figure 2. ANFs presented a one-dimensional fibrous structure, with a fiber diameter ranging from 10 to 30 nm, consistent with other research findings [1,13]. After modification, T-ANFs still maintained their fibrous structure but exhibited a rougher surface compared to ANFs. Furthermore, the fiber diameter of T-ANFs noticeably increased to 20–50 nm, which might have resulted from the introduction of methyl methacryloyloxy groups onto the fiber surface. Dong et al. [33] also observed a significant increase in the diameter of ANFs after modification with 1-bromododecane, consistent with the results of this study.

**Figure 3:**
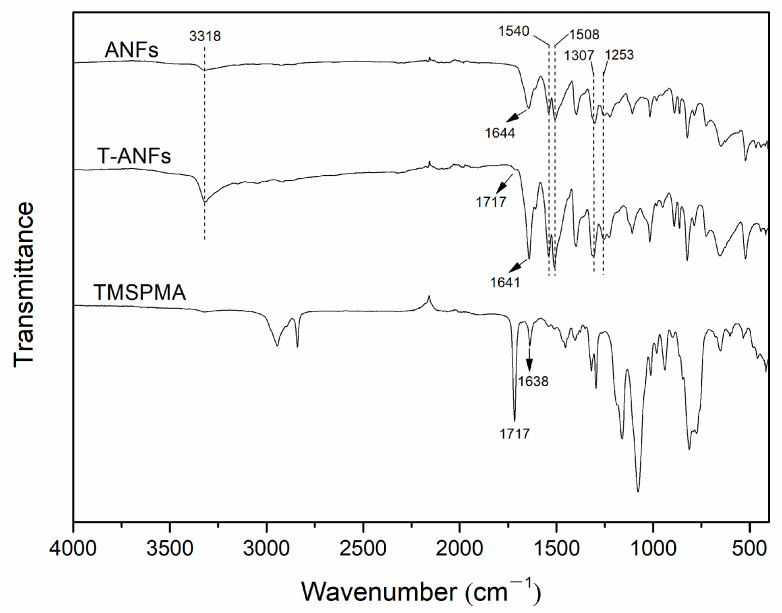
FTIR spectra of the TMSPMA, ANFs, and T-ANFs.

Furthermore, the chemical structure of ANFs and T-ANFs was characterized using FTIR (Figure 3). In the FTIR spectrum of ANFs, characteristic peaks of poly-p-phenylene terephthalamide (PPTA) were observed, namely, the stretching vibrations of N-H and C=O at 3318 and 1644 cm^−1^, the coupled bending vibrations of C-N and N-H at 1540 and 1253 cm^−1^, and the stretching vibrations of C=C and Ph-N on the aromatic ring at 1508 and 1307 cm^−1^ [8,11]. In the FTIR spectrum of T-ANFs, these characteristic peaks were still present, indicating that the main chain chemical structure of ANFs remained intact after modification. In the FTIR spectrum of TMSPMA, the stretching vibrations of C=O and C=C in the methyl methacryloyloxy groups corresponded to peaks at 1717 and 1638 cm^−1^, respectively [16,34]. In the FTIR spectrum of T-ANFs, a peak corresponding to the stretching vibration of C=O in the methyl methacryloyloxy groups appeared at 1717 cm^−1^. Additionally, the peak corresponding to the stretching vibration of C=O in the main chain shifted from 1644 cm^−1^ in ANFs to 1641 cm^−1^ in T-ANFs, and its intensity significantly increased. This result suggests that the combination of the stretching vibration peak of C=C in the methyl methacryloyloxy groups and the stretching vibration peak of C=O in the main chain of ANFs contributed to the observed peak. These results indicate that the surface of modified ANFs has successfully grafted methyl methacryloyloxy groups.

### 3.2. Curing Kinetics Analysis

**Figure 4:**
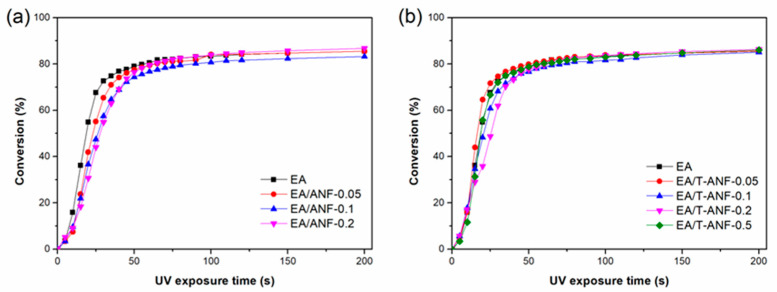
Conversion versus time kinetic curves of the (**a**) EA/ANF and (**b**) EA/T-ANF nanocomposite films.

Real-time FTIR was used to study the effects of ANFs and T-ANFs on photopolymerization kinetics, and Figure 4 shows the C=C double bond conversion rate curves at different irradiation times. As seen from the figure, all curves exhibit a trend of rapid initial increase followed by a plateau with increasing UV irradiation time, indicating that photopolymerization reactions proceed rapidly in the initial stage of irradiation, and some C=C bonds are trapped and eventually unable to undergo polymerization reaction as crosslinked networks of the polymer are formed, ending the photopolymerization reaction. From Figure 4a, it can be seen that with increasing ANFs content, the initial photopolymerization rate decreases, mainly due to the strong absorption of UV light by ANFs [30,35,36], which hinders the absorption of UV light by the photoinitiator in the initial stage of photopolymerization reaction. However, the final double bond conversion rates of all samples remained above 83%, indicating that the addition of ANFs had no significant effect on the polymerization quantum yield. From Figure 4b, it can be observed that when the conversion rate is less than 30%, the photopolymerization rates of all samples are basically the same. However, when the conversion rate exceeds 30%, the photopolymerization rate shows a trend of first decreasing and then increasing with the increase of T-ANF content. This is due to the competitive absorption of UV light by T-ANFs, causing a decrease in the photopolymerization rate on the one hand, and the provision of more C=C double bond active groups, leading to an increase in the photopolymerization rate on the other hand. Therefore, the two effects offset each other in the initial stage of the reaction, resulting in a basically unchanged photopolymerization rate. When the conversion rate exceeds 30% for samples with T-ANF content lower than 0.2 wt%, the active groups in T-ANF have already undergone polymerization reactions, thus, its competitive absorption of UV light becomes dominant. Consequently, the photopolymerization rate gradually decreases with the increase of T-ANF content. As for the sample with a T-ANF content of 0.5 wt%, due to its ability to provide more C=C double bond active groups, it can still provide more opportunities for contact with the surrounding photoinitiator when the conversion rate exceeds 30%, leading to an increase in the photopolymerization rate. In addition, the impact of T-ANFs on the final double bond conversion rate was insignificant, and the final double bond conversion rates of EA/T-ANF nanocomposite films remained above 85%.

### 3.3. Mechanical Properties of EA/ANF and EA/T-ANF Nanocomposite Films

**Figure 5:**
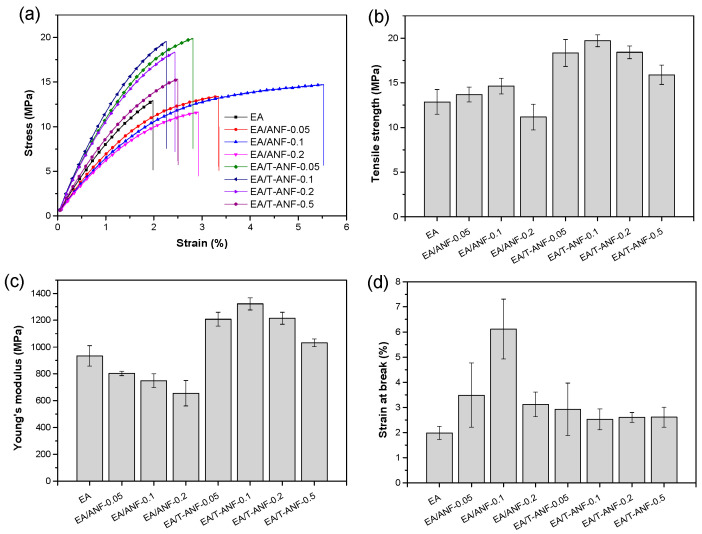
(**a**) Typical stress-strain curves, (**b**) tensile strength, (**c**) Young’s modulus, and (**d**) elongation at break of the EA/ANF and EA/T-ANF nanocomposite films.

**Figure 6:**
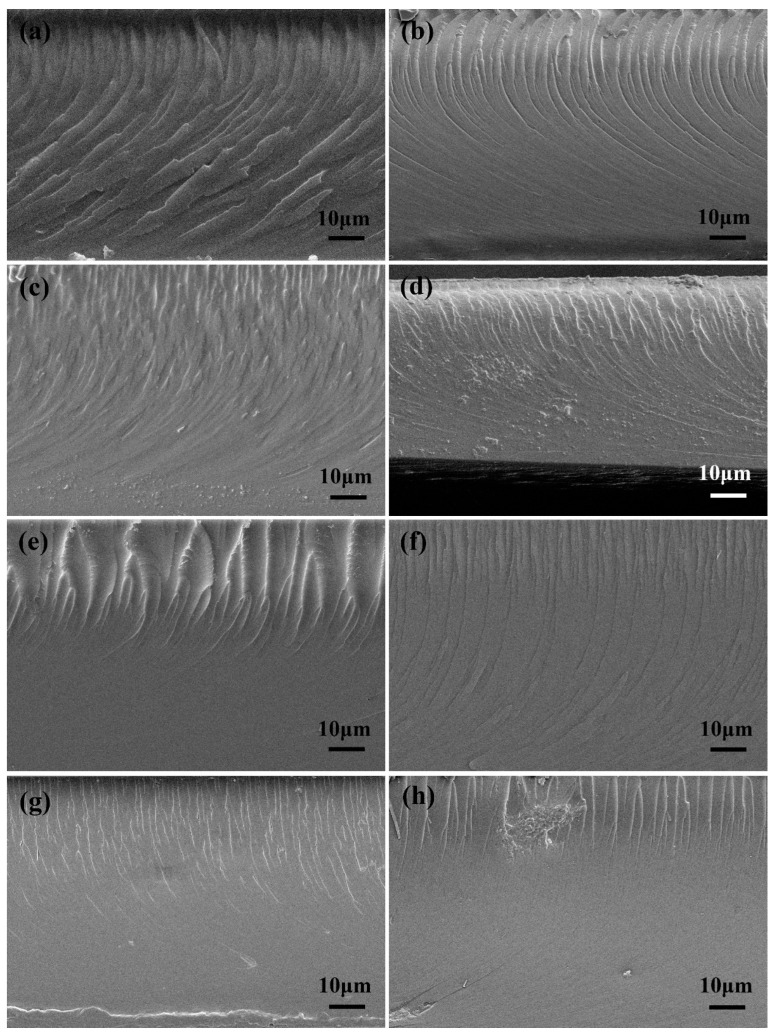
SEM images of the fractured surfaces of (**a**) EA, (**b**) EA/ANF-0.05, (**c**) EA/ANF-0.1, (**d**) EA/ANF-0.2, (**e**) EA/T-ANF-0.05, (**f**) EA/T-ANF-0.1, (**g**) EA/T-ANF-0.2 and (**h**) EA/T-ANF-0.5.

The effects of ANFs and T-ANFs on the mechanical properties of the nanocomposite films were investigated using tensile testing (Figure 5). In order to further study their dispersion morphology and reinforcement mechanism, the cross-sectional morphology of the films was characterized by SEM (Figure 6). As shown in Figure 5b,d, the tensile strength and elongation at break both exhibited a trend of initially increasing and then decreasing with increasing ANF content. This is mainly attributed to the excellent mechanical properties and flexibility of ANFs, which can absorb more energy before the fracture of the films. However, as shown in Figure 6a–d, with the increase in ANF addition, the fracture surface became rougher, indicating a decrease in the dispersion performance of ANFs in the polymer matrix. In Figure 6d, the formation of micron-scale aggregates in the films can be observed, which leads to stress concentration during the tensile process and ultimately deteriorates the mechanical properties. Furthermore, as shown in Figure 5c, the Young’s modulus gradually decreased with increasing ANFs content. This is mainly due to the addition of ANFs reducing the degree of cross-linking in the polymer matrix, resulting in a decrease in the film stiffness.

Compared to ANFs, T-ANFs have a significantly different effect on the mechanical properties of the films. Firstly, the addition of T-ANFs leads to a substantial increase in both tensile strength and Young’s modulus of the films. For instance, the pure EA film exhibits a tensile strength and Young’s modulus of 12.9 and 933.8 MPa, respectively, while the EA/T-ANF-0.1 film shows an increase of 52.7% and 41.6% in tensile strength (19.7 MPa) and Young’s modulus (1322.2 MPa), respectively, compared to the pure EA film. This can be attributed to two main factors. On the one hand, as depicted in Figure 6e–h, at the same additive level, the fracture surface of the EA/T-ANF nanocomposite films appears smoother than that of the EA/ANF nanocomposite films, indicating a more uniform dispersion of T-ANFs within the polymer matrix facilitated by the methyl methacryloyloxy groups on the surface of T-ANFs. On the other hand, T-ANFs can participate in photopolymerization and chemically bond with the polymer matrix, leading to a significant enhancement in cross-linking density and stiffness. These two factors enable T-ANFs to better absorb energy prior to fracture, avoiding deterioration of the mechanical properties caused by stress concentration due to aggregation. As shown in Figure 6h, significant aggregates on the fracture surface of the film only appear when the T-ANFs addition reaches 0.5 wt%, which is also the reason for the slight decrease in mechanical properties. It is important to note that the improvement in fracture elongation achieved by T-ANFs is much lower compared to ANFs, primarily due to the increased interfacial cross-linking density and rigidity resulting from T-ANFs addition.

Other studies have also enhanced the mechanical properties of epoxy acrylate-based UV-curable coatings by adding fillers. For instance, Çanak et al. [37] found that the addition of 10 wt% boron increased the Young’s modulus and tensile strength of the films by 27.9% and 90.0%, respectively. Yan et al. [38] investigated the influence of graphene on the tensile properties of hyperbranched silicone epoxy acrylate resin-based UV-curable coatings, and the results indicated that the addition of 0.75 wt% graphene increased the tensile strength of the films by approximately 57%, but led to a significant decrease in elongation. Guan et al. [39] prepared hyperbranched polyester polythiol (H20-SH) and incorporated it into epoxy resin-based UV-curable coating systems, demonstrating that the addition of 11 wt% H20-SH could increase the tensile strength of the coatings by about 19%. In comparison to the aforementioned studies, our research is able to enhance the comprehensive performance of the films, including tensile strength, Young’s modulus, and elongation at break, by incorporating a minimal amount of fillers.

## 4. Conclusions

In this study, TMSPMA-functionalized ANFs were synthesized and incorporated into UV-curable epoxy acrylate-based coatings to prepare a series of nanocomposite films using UV curing technology. Compared to ANFs, the surface of T-ANFs became rougher, and the fiber diameter increased significantly. Additionally, FTIR spectra confirmed the successful grafting of methyl methacryloyloxy groups onto the surface of the ANFs. The presence of both ANFs and T-ANFs led to competitive absorption of UV light, resulting in a slight decrease in the initial curing rate but had minimal impact on the final double bond conversion rate, which remained above 83%. The addition of ANFs increased the tensile stress and elongation at break of the films, but it also reduced the cross-linking density of the polymer matrix, leading to a decrease in stiffness and Young’s modulus of the films. In contrast, the reactive groups on T-ANFs facilitated more uniform dispersion within the polymer matrix and participated in the UV-curing reaction, leading to a significant improvement in tensile stress and Young’s modulus. This study investigated the interaction mechanism between functionalized ANFs and the polymer matrix, providing new insights for enhancing the mechanical properties of UV-curable epoxy acrylate-based coatings. In the future, further research can be conducted on how to achieve uniform dispersion of aramid nanofibers, even at higher concentrations, in order to better enhance the mechanical properties of the films.

## Data Availability

Data are contained within the article.
